# Analysis of the Chemical and Physical Environmental Aspects that Promoted the Spread of SARS-CoV-2 in the Lombard Area

**DOI:** 10.3390/ijerph18031226

**Published:** 2021-01-29

**Authors:** Roberto Dragone, Giorgio Licciardi, Gerardo Grasso, Costantino Del Gaudio, Jocelyn Chanussot

**Affiliations:** 1 Istituto per lo Studio dei Materiali Nanostrutturati sede Sapienza, Consiglio Nazionale delle Ricerche, P.le Aldo Moro 5, 00185 Rome, Italy; 2 Gipsa-lab, Grenoble Institute of Technology, 38031 Grenoble, France; giorgio-antonino.licciardi@grenoble-inp.fr (G.L.); jocelyn.chanussot@grenoble-inp.fr (J.C.); 3 Fondazione E. Amaldi, Via del Politecnico, 00133 Rome, Italy; costantino.delgaudio@fondazioneamaldi.it

**Keywords:** COVID-19, particulate matter, meteorology, public health, epidemiology

## Abstract

Recent works have demonstrated that particulate matter (PM) and specific meteorological conditions played an important role in the airborne transmission of the SARS-CoV-1 and MERS-CoV. These studies suggest that these parameters could influence the transmission of SARS-CoV-2. In the present investigation, we sought to investigate the association between air pollution, meteorological data, and the Lombardy region COVID-19 outbreak caused by SARS-CoV-2. We considered the number of detected infected people at the regional and provincial scale from February to March 2020. Air pollution data were collected over the Lombardy region, nominally, sulphur dioxide, ammonia, nitrogen dioxide, nitrogen monoxide, carbon monoxide, ozone, and suspended particulate matter measuring less than 10 μm (PM10) and less than 2.5 μm (PM2.5). Meteorological data have been collected over the same region for temperature, relative humidity, and wind speed. In this work, we evaluated the combined impact of environmental pollutants and climate conditions on the COVID-19 outbreak. The analysis evidenced a positive correlation between spatial distribution of COVID-19 infection cases with high concentrations of suspended particulate matter and a negative relationship with ozone. Moreover, suspended particulate matter concentration peaks in February correlated positively with infection peaks according to the virus incubation period. The obtained results suggested that seasonal weather conditions and concentration of air pollutants seemed to influence COVID-19 epidemics in Lombardy region.

## 1. Introduction

With each new day, the unprecedented impact of the new coronavirus (SARS-CoV-2) pandemic on Europe’s health and economy becomes more evident. Even if we are getting better at coping with this virus as we learn more about its behavior, a complete knowledge of its infections is still an open issue. The COVID-19 outbreak indicates the relatively efficient human-to-human transmission of SARS-CoV-2. In particular, based on ongoing studies, the transmission of the virus is likely to occur through respiratory secretions in the form of droplets (>5 μm) and aerosols (<5 μm). In this regard, it is worth considering the stability of the virus in air, because it may directly affect its transmission. Based on particular atmospheric conditions, the virus particles in the air may remain active long enough after being expelled from one host to infect a new host. It has been demonstrated that airborne transmission played an important role in the extraordinary transmission of the SARS-CoV-1 and MERS-CoV [1,2,3].

Since January 2020, millions of people worldwide contracted the virus with an average mortality rate between 2% and 5% [4]. However, some areas of the world presented a contagion rate higher than the average. The main cause of these anomalies can be related to differences in the national health services and to specific management protocols of the crisis. The Italian region of Lombardy belongs to these areas accounting for more than 40% of the infections of the whole country and presenting a 24-h infection growth rate higher than the rest of the Italian regions [5]. As the anomalies between regions are within the same country, it is unlikely that the main cause of these anomalies could be ascribed to strong differences in the health services.

A possible origin of these anomalies could be related to specific conditions that may facilitate aerial transmission through aerosolized SARS-CoV-2. The concentration and size of viral bioaerosol particles strongly affect probability that a viral infection becomes established. The minimal infective doses of SARS-Cov-2 for human is currently unknown, accounting for all possible exposure routes, although some data for a non-human primate model (Macaca mulatta) are available [6,7]. The primary mode of transmission of SARS-CoV-2 appears to be the direct contact of mucous membrane of the nose, mouth, and eyes with infectious respiratory droplets and/or through exposure to fomites. Concerning the potential tropism of the here considered coronavirus, a possible explanation for its high efficiency can be related to the ACE2 cellular receptor involved in SARS-CoV-2 binding, which is expressed in respiratory, corneal, and intestinal epithelial cells [8]. Hence, the risk of transmission of SARS-CoV-2 through human conjunctival epithelium should be properly addressed, and the use of adequate personal protective equipment, like eyewear safety glasses, should be encouraged [9].

In general, aerial transmissions of respiratory infections can be divided in two different groups according to the size of the droplets: transmission occurs when a person is in close contact with someone else having respiratory symptoms and then is at risk of having the mucosae or conjunctiva (eyes) exposed to potentially infective respiratory droplets, having size >5–10 μm. Airborne transmission refers to the presence of microbes, which are generally considered to be particles <5 μm in diameter that can remain in the air for long periods of time and be transmitted to others over distances greater than 1 m [10,11,12,13,14]. Concerning the SARS-CoV-2 virus, there is evidence that the primary transmission route is through respiratory droplets [15]. However, previous studies suggested the correlation between air pollution and increased respiratory symptoms, hospital admissions, and viral respiratory infections [16,17,18,19,20]. Although there is no evidence yet that air pollution and meteorology are playing a role in spreading the SARS-CoV-2, recent research suggests that its spread could be affected by specific environmental conditions [21,22].

From this point of view, particulate matter, belonging to the atmospheric aerosols, have been identified in the literature as an important means of virus transmission in humans. In the literature, many authors reported the isolation of enteric viruses from aerosols generated from different sources [23,24,25,26,27]. The permanence of virus in aerosol can vary from few hours up to several days [28]. In general, the most important factors affecting the stability of viruses in the aerosol state are temperature, pH, relative humidity, moisture content, size of the aerosol particle, composition of the suspending medium, sunlight exposure, air quality, and virus type. However, the basis of virus inactivation in aerosols is still poorly understood, and there is not a general rule to describe this effect [29,30,31,32,33,34,35,36].

Virus persistence in aerosols can also be affected by solutes in the suspending media of the aerosol. Salts and proteins may provide protection against dehydration and thermal inactivation of aerosolized viruses [37,38].

Aerobiological aspects related to the interaction between PM with viral bioaerosols as well as the possible effects of such interaction on the stability of airborne viruses are not frequently considered in research about PM air pollution [39,40]. Although the composition of PM could have an important role in the spread of the virus, in most studies it is generally not reported. In addition, the composition of PM can change between different places, depending on sources and processes that produce PM, like vehicular transport, industrial activities, and domestic heating. Thus, further studies are needed also to determine how composition of PM can affect interaction with viral particles (through noncovalent bonds) and their stability and promote the diffusion of viral particles.

SARS-CoV-2 persistence in air and the protective effects of relative humidity and ambient temperature have not been investigated yet. However, it has been pointed out that SARS-CoV-2 is similar to SARS-CoV viruses [41], and, in this regard, it was reported that at 4 °C SARS-CoV-1 persisted for as long as 28 days in aerosols. Concerning the relationship between relative humidity and inactivation of virus, a greater protective effect has been demonstrated at low RH (20%) and high RH (80%) rather than at moderate RH (50%) [42].

These studies were conducted in a protected environment; thus, the effective behavior of the virus in an open environment should be assessed. The aim of this study is to investigate the possible correlation between meteorological conditions and air pollutants with the spread of SARS-CoV-2 infections in the Lombardy region. In particular, while temperature and relative humidity will be considered as a descriptor of persistence of virus in aerosols, there are other parameters that may influence the stability of the virus in the air, such as particulate matter (and air pollutants in general), wind speed, and net atmospheric acidity.

## 2. Materials and Methods

### 2.1. Cases Identification

In this study, we analyzed epidemiological data provided daily from the Istituto Superiore di Sanità (ISS) [43] and Protezione Civile [44], reporting the geographic distribution, updated daily, across the 12 Lombardy provinces acquired from 24 February 2020 (the first day the coronavirus has been identified in Italy), to 31 March 2020.

This temporal interval has been chosen taking into account the different phases of the lockdown that have been applied in Italy. Indeed, an initial lockdown began on 21 February in ten municipalities of the Lodi province (Lodi, Italy). Subsequently, on 8 March, the lockdown was expanded to most of the whole Lombardy region and several municipalities of Piedmont, Veneto, Emilia-Romagna, and Marche. On 9 March, the lockdown was extended to the whole Italian country; however, only on 21 March was a real lockdown applied by shutting down all non-necessary business and industries.

In order to carry out the proposed analysis, we considered the number of people affected by coronavirus categorized by region and province of residence. In particular, for this study, we considered:

- The daily incidence rate, defined as the number of new cases over the considered population, and used to measure the frequency with which the disease occurs daily.

$$\mathrm{Incidence}=\frac{\mathrm{New}\;\mathrm{daily}\;\mathrm{cases}}{\mathrm{Population}\;\mathrm{at}\;\mathrm{risk}}$$

- The daily prevalence rate, defined as the number of infected cases over the considered population sampled every day, and used to evaluate the spreading of the virus

$$\mathrm{Prevalence}=\frac{\mathrm{total}\;\mathrm{number}\;\mathrm{of}\;\mathrm{cases}}{\mathrm{Population}\;\mathrm{at}\;\mathrm{risk}}$$

- The growth factor, defined as the factor by which a quantity multiplies itself over time:

$$\mathrm{Growth}=\frac{\mathrm{Every}\;\mathrm{day}’s\;\mathrm{new}\;\mathrm{cases}}{\mathrm{new}\;\mathrm{cases}\;\mathrm{of}\;\mathrm{the}\;\mathrm{previous}\;\mathrm{day}}$$

This means that a growth factor having a value between 0 and 1 represents a sign of decline, while values above 1 indicate an increase. For example, a growth factor of 1.1 represents a quantity growing by 10%. A growth factor constantly above 1 represents an exponential growth.

### 2.2. Meteorological Data

Meteorological data for the geographic area of Lombardy region were recorded from Lombardy Meteorological Stations. Data relative to temperature (Celsius degrees), relative humidity (%), and wind speed (Km/h) were recorded daily for each province within Lombardy region during the period between 1 February 2020, and 31 March 2020. We decided to take into account this temporal period based on the average incubation period that was estimated to be between 5.1 and 11.5 days, with some cases overpassing 14 days [45].

### 2.3. Air Pollutants Data

Analogously to the meteorological data, we collected daily concentration data from 1 February 2020 to 31 March 2020 over the Lombardy region with a spatial resolution of 10 km. The data were collected through the Copernicus Atmosphere Monitoring Service (CAMS), implemented by the European Centre for Medium-Range Weather Forecasts (ECMWF) as part of the Copernicus Programme. Copernicus is the European Union Earth Observation Programme offering several services based on satellite Earth Observation and ground-based data. CAMS provides consistent and quality-controlled information related to air pollution everywhere in the world.

The following air pollutants concentrations (μg/m^3^) were selected: sulfur dioxide (SO_2_), nitrogen dioxide (NO_2_), nitrogen monoxide (NO), carbon monoxide (CO), ozone (O_3_), ammonia (NH_3_), and levels of suspended particles with an aerodynamic diameter less than 10 and 2.5 μm (PM10, PM 2.5).

To estimate the possible neutralization of the principal acidic gasses (NO_2_, NO, and SO_2_) by NH_3_, net atmospheric acidity (NAA) was calculated as follows [46]:

NAA = [NO_2_]_gas_ + [NO]_gas_ + 2[SO_2_]_gas_ − [NH_3_]_gas_ (1)

The micromolar concentrations reported in (1) have been calculated as the ratio of the concentration values and the molecular weight of each gasses (30.01, 46.01, 64.06, and 17.03 for NO, NO_2_, SO_2_, and NH_3_ respectively).

With regard to particulate matter, it has been recognized that precipitations are of the primary natural processes to reduce PM in most areas. The scavenging effects of precipitation on PM is mainly manifested by wet deposition and wet removal [47,48]. Moreover, it has been demonstrated that the impacts of wind and precipitation on different-sized PM concentrations are significantly different [49,50]. Indeed, it has been pointed out that the fine PM concentrations decreased gradually with the increase of wind speed, while coarse PM concentrations would increase due to dust resuspension under strong wind. More in general, low-speed wind or calm conditions will facilitate the accumulation of the fine particles, but high-speed wind may be in favor of the PM resuspension and thus increase coarse PM concentration [51].

### 2.4. Data Analysis

As stated before, the propagation of aerosolized viruses through the air can be affected by several factors, with temperature and humidity being two of the most prominent environmental elements. Moreover, it has been evidenced that exposure to air pollutants, in particular to PM2.5 and PM10, is associated with hospitalization due to viral infection.

In this study, we considered both qualitative and quantitative evaluations, conducting two different analysis of the possible correlation between environmental parameters and the anomalous spreading of SARS-CoV-2 in the Lombardy region. In the first instance, we investigated the spatial correlation between the prevalence of infected cases and concentration of air pollutants. For this analysis, we compared the spatial distribution of total infected people on provincial basis obtained on 31 March, with the concentration of PM2.5, PM10, CO, and NH_3_ averaged during the period between 1 February and 31 March. In order to evaluate this correlation, we considered the Pearson correlation coefficient.

On a second instance, we conducted a time-series analysis on the prevalence of infected cases occurring in the Lombardy region, to verify the hypothesis that exposure to high concentration of air pollutants in combination with particular environmental conditions may be associated with the insurgence of coronavirus infection. The analysis has been carried out considering each province separately, investigating the presence of anomalies in the growth factor, in order to detect possible correlation with air pollutants concentration.

Regarding the air pollutants, aside from identifying the peaks of PM10 and PM2.5 over the investigated regions, we also investigated the persistence time of particulate matter during the period between 24 February 2020 and 31 March 2020, for each province. With regards to PM, we also considered the occurrence of precipitations that may considerably reduce their concentrations, and wind speed, which may have different effects depending on particulate size. In particular, we investigated the occurrence of strong wind and precipitations after a long period of persistence of particulate matter over the same area. The analysis has been carried out in a heuristic way by summing up the daily occurrence of the above-mentioned events.

## 3. Results

Analyzing the period between 24 February 2020 and 31 March 2020, it emerged that more than 63% of the 42,283 infected people registered in the whole Lombardy region were concentrated in three provinces, Milan, Bergamo, and Brescia, accounting for 8911, 8803, and 8367 cases, respectively. More in particular, while at national level the average ratio between infected cases and population was about 0.21%, in Lombardy this ratio doubled to 0.42%, with Bergamo (0.79%), Brescia (0.66%), Cremona (1.08%), and Lodi (0.92%) having the highest contagion rates.

### 3.1. Spatial Analysis

**Figure 1:**
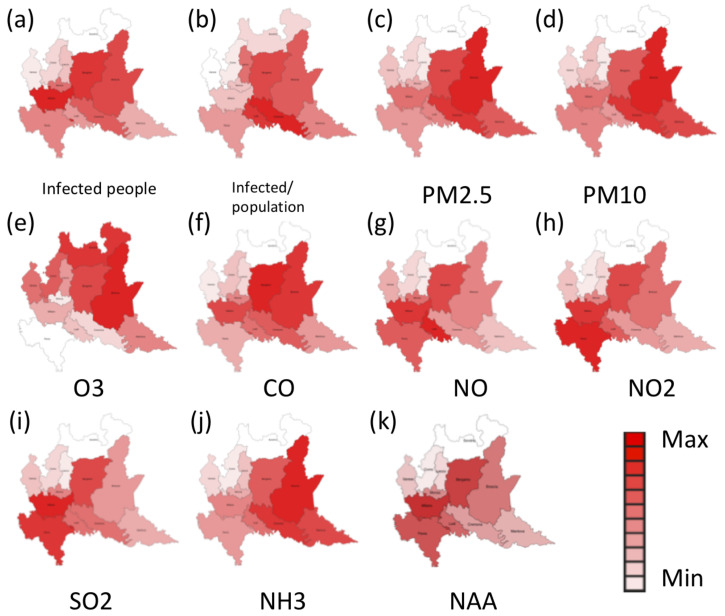
Distribution over the different provinces of Lombardy of (**a**) total number of infected people, (**b**) daily prevalence rate (Infected /population), (**c**) average concentration of particulate matter 2.5 (PM 2.5), (**d**) average concentration of particulate matter 10 (PM10), (**e**) ozone (O_3_), (**f**) carbon monoxide (CO), (**g**) nitric oxide (NO), (**h**) nitrogen dioxide (NO_2_), (**i**) sulfur dioxide (SO_2_), (**j**) ammonia (NH_3_) and (**k**) net atmospheric acidity (NAA) during the considered time period.

In a first analysis, we investigated the spatial correlation between the prevalence of infected people and concentration of air pollutants for each province. The Pearson correlation coefficients ρ, measuring the linear correlation between the average concentrations of air pollutants and the prevalence of infected people, reported in Table 1, show a good correlation with almost all air pollutants. In particular, CO and NH_3_ gasses present the highest values for ρ, while O_3_ presents the weakest correlation. This can be also deduced by qualitatively analyzing the maps reported in Figure 1.

These results suggest that PM10, PM2.5, NH_3_, and CO may have some correlation with the spread of the SARS-CoV-2 virus in Lombardy.

Considering the net atmospheric acidity as an important factor for the persistence of virus in the air, we also considered the correlation of the number of infected people with the NAA, as reported in Table 1.

### 3.2. Temporal Analysis

A more complex analysis has been carried out on a time series of the evolution of the virus infection and the possible correlation with meteorological and air pollutants data. In particular, we focused on those provinces that presented anomalies in the contagion rates. In this regard, it is important to consider also the different phases of the lockdown that restricted the movement of the population except for necessity, work, and health circumstances. The first lockdown took place on 21 February in eleven municipalities in Lodi province, where 16 people were infected. On 8 March, a soft lockdown was extended to all the Lombardy region and part of Veneto, Emilia Romagna, Marche, and Piedmont regions, affecting more than 16 million of people. However, on 9 March, the lockdown was extended to all of the Italian country, and starting from 11 March, the lockdown has been tightened, closing down most of the commercial business. These measures were taken to reduce the contagion growth rate. Thus, it was expected that the growth rate stabilized after a certain period, which should coincide with the 14 days of virus incubation.

**Figure 2:**
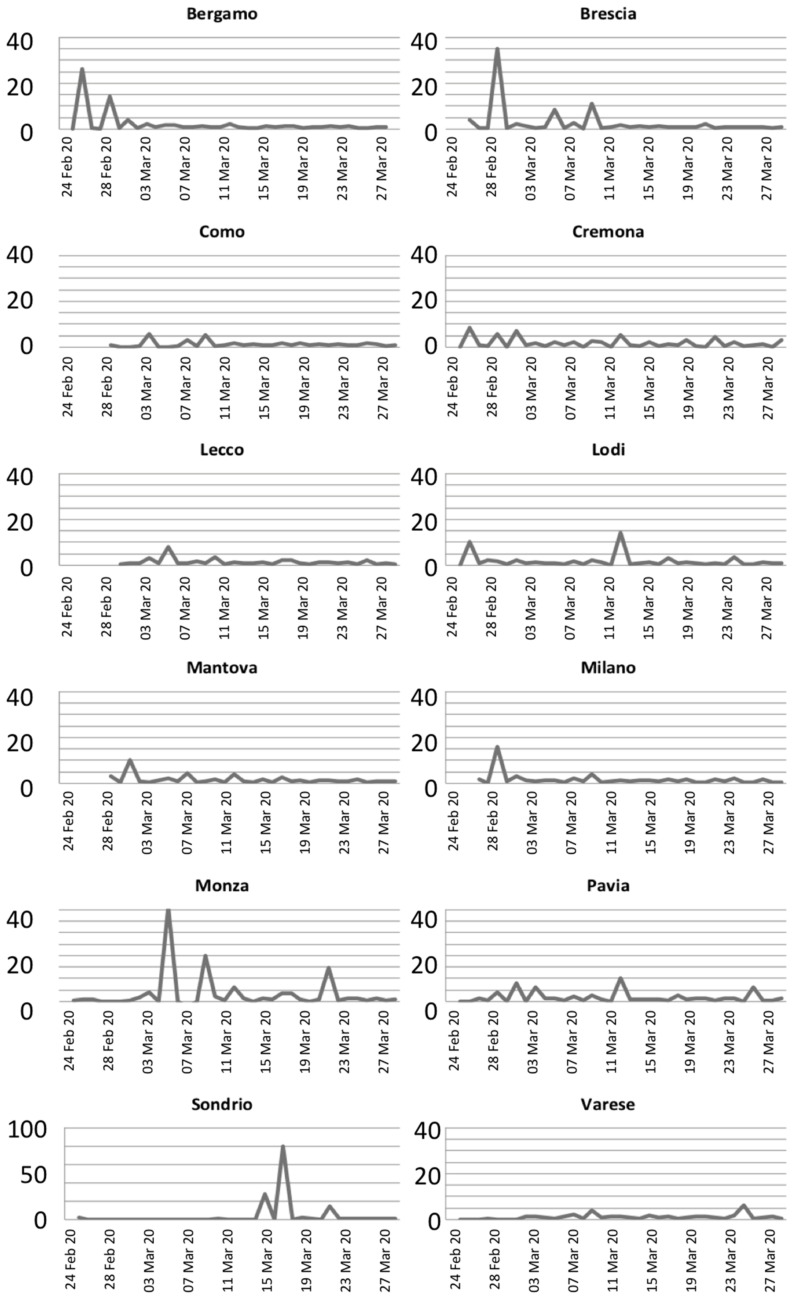
Growth rates of the infection on divided per provinces.

The graphs shown in Figure 2 report the evolution of the growth rates for each province through time. This parameter measures the disease’s potential at the initial stage of virus spread; the growth rate can increase rapidly and present several spikes. However, after the activation of countermeasures, such as screening and quarantines, the growth rate stabilizes and should present a regular trend.

As expected, in most of the provinces, the growth rate presented regularized values around 1.5 after the initial lockdown in the Lombardy region, on 21 February. However, some anomalies in the growth rates can be noted in Bergamo, Brescia, Milano, Monza, and Sondrio after the application of countermeasures. For the latter province, the anomaly is due to a cumulative report of infected people on 18 March, while for the other provinces, the growth rates indicate anomalies in the contagion of the virus.

**Figure 3:**
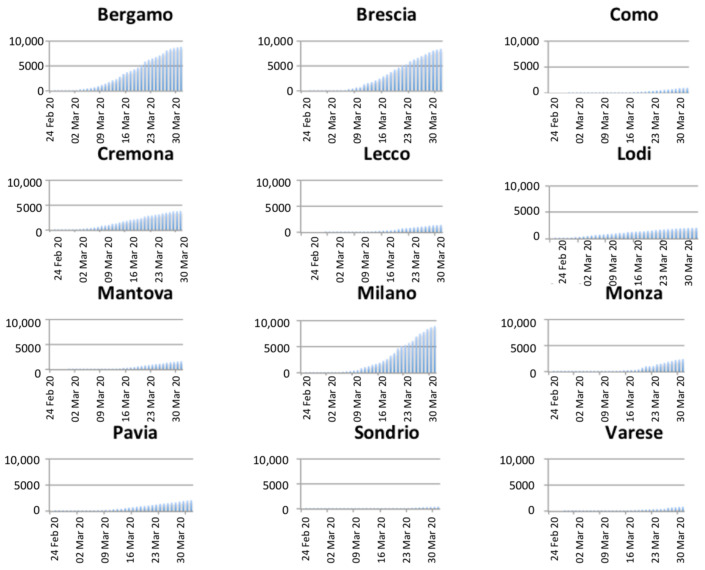
Temporal evolution of the number of infected people per province.

Moreover, graphs in Figure 3, reporting the evolution of infected people through time, evidence exponential growths for the provinces of Bergamo, Brescia, and Milan, while it is linear for the other provinces.

**Figure 4:**
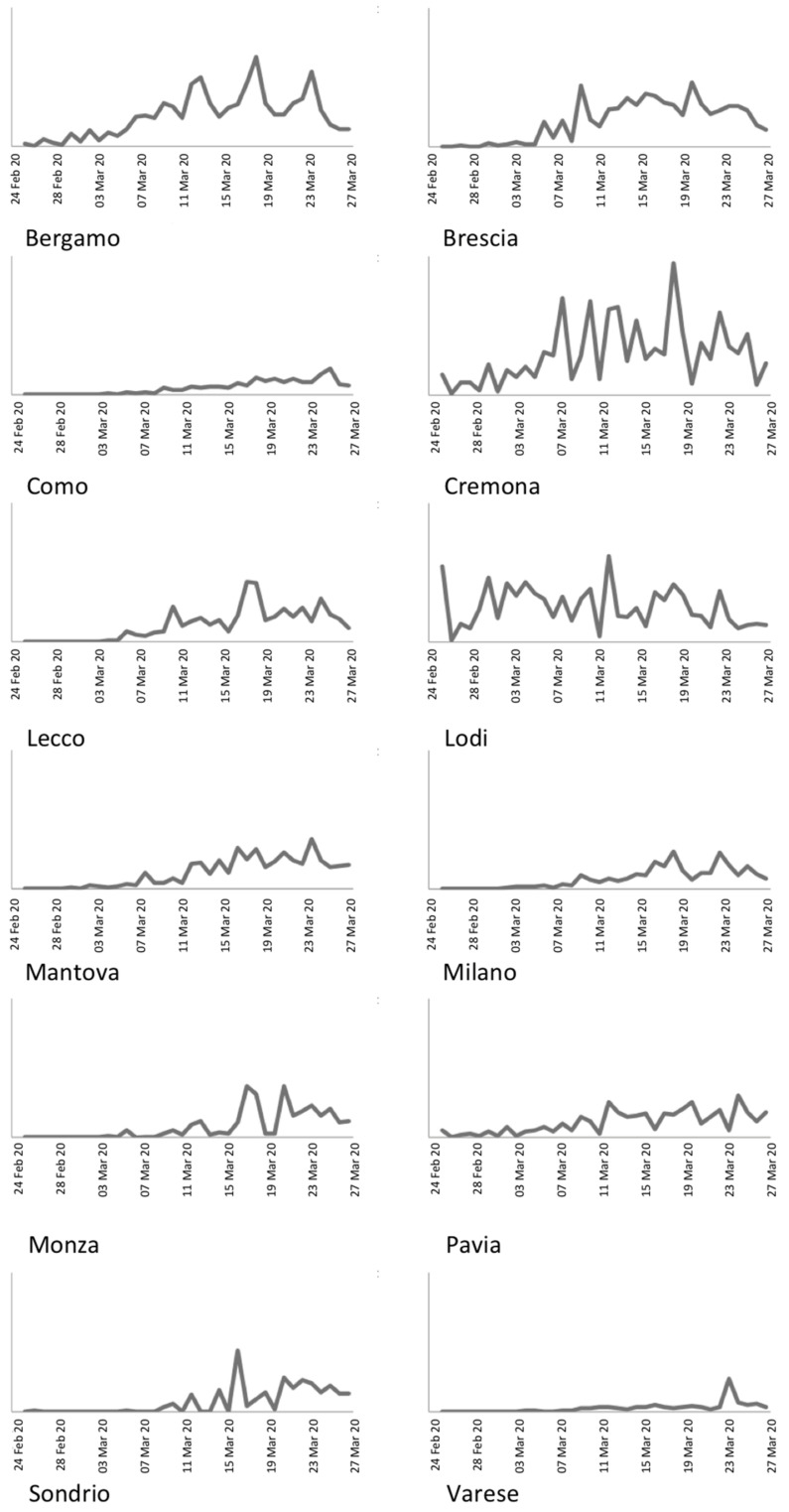
Daily incidence rate for the Lombardy provinces.

Another important qualitative analysis can be carried out by inspecting the daily incidence and prevalence rates. After the adoption of countermeasures to reduce the contagion rate, it is expected that both rates stabilize to constant value, without strong variations. However, Analyzing Figure 4, reporting the daily incidence rate, it can be noted that the province of Bergamo presents three pronounced peaks on 15, 21, and 27 March. Similar patterns can be identified also in Brescia (11, 18, and 23 March), Milano (11, 21, and 27 March), and Lecco (12, 20, and 28 March).

**Figure 5:**
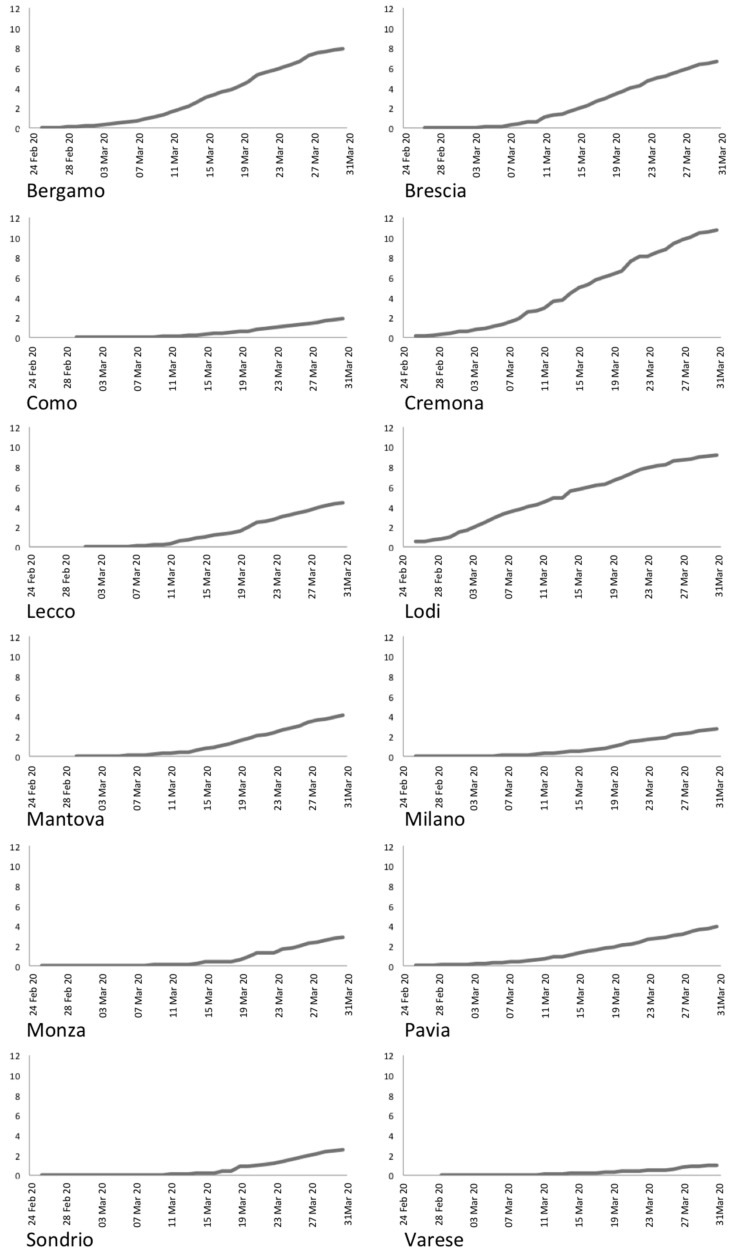
Daily prevalence rate for the Lombardy provinces.

Another important issue can be noted upon accurate analysis of graphs depicted in Figure 5. As expected, Lodi and Cremona present increasing prevalence rates from the first days, being the first hotbeds of the virus infection in Italy. On the other hand, the other provinces present changes in the trends between 9 March and 15 March, depending on the province. This is particularly evident for the provinces of Bergamo and Brescia, and less evident for Mantova and Monza. This suggests a possible outbreak between 25 February and 2 March, according to the virus incubation period, in any cases a week later than the first confirmed case in Italy.

**Figure 6:**
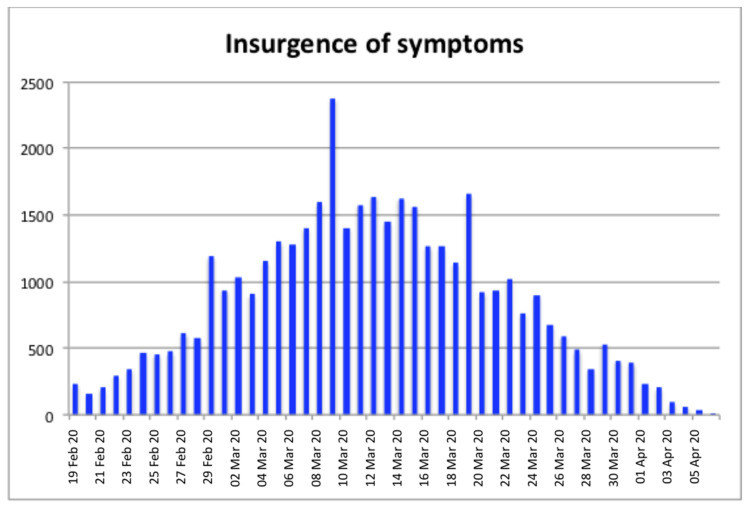
Number of infected people organized by date of insurgence of the first symptoms.

In Figure 6, the insurgence of the symptoms is reported for the whole Lombardy region. It is worth noting that the dates of the first symptoms are disconnected from the dates of hospitalization that could be happened several days after. Additionally, in this case, it is possible to note the presence of three anomalous peaks on 29 February and 9 and 19 March, respectively.

Assuming the transmission of virus through air, we analyzed the occurrence of all the conditions that could be favorable for the persistence of virus in the air: (i) meteorological conditions of relative humidity around 80 ± 5% and ambience temperature around 6 ± 2 °C and (ii) atmospheric conditions of high amounts of particulate matter in the air, i.e., above the daily limit of 30 μg/m^3^ as a possible threshold. Another important condition that could facilitate the survival of virus in air is related to the acidity of the atmospheric gases. In this case, we considered the balance between NH_3_ with NO, NO_2_, and SO_2_; if NH_3_ concentration is higher than NO + NO_2_ + 2SO_2_, then the net atmospheric acidity is supposed to preserve the vitality of the virus (see Discussion section).

**Figure 7:**
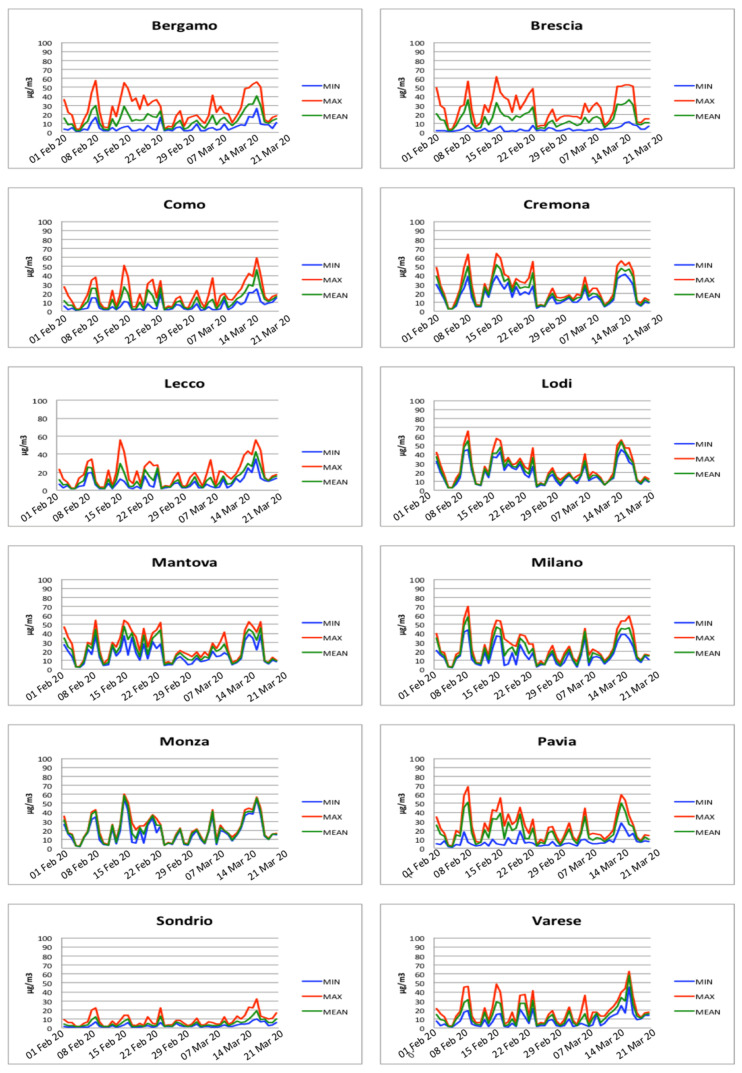
PM2.5 concentrations measured for each province.

**Figure 8:**
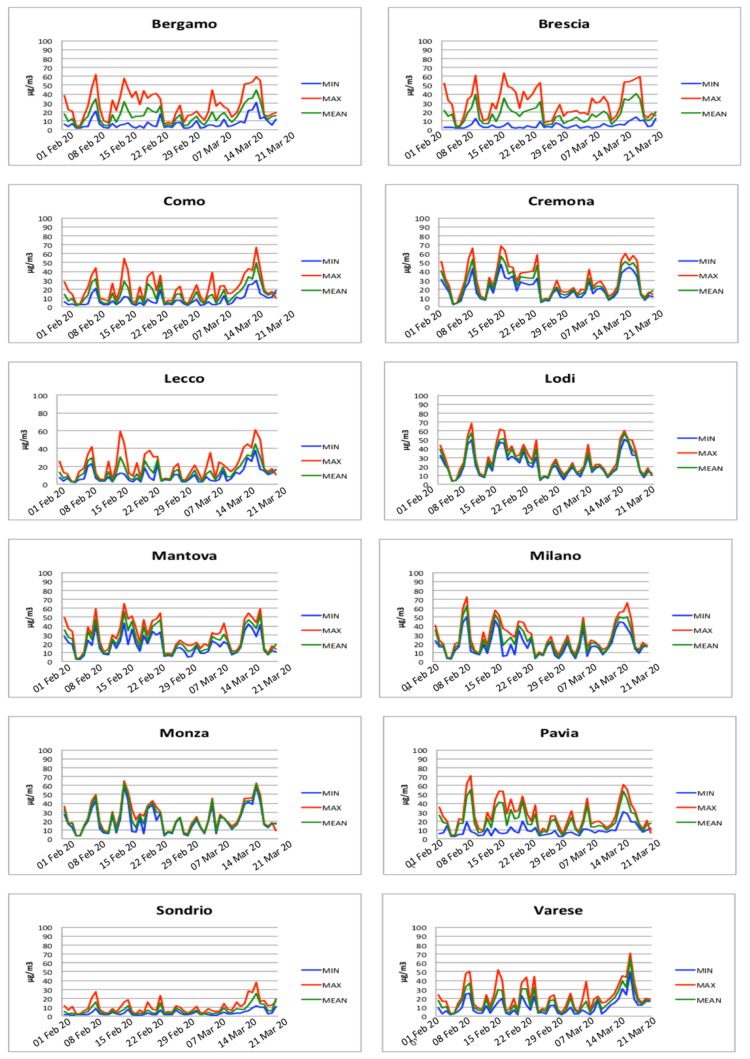
PM10 concentrations measured for each province.

Through the analysis of temporal evolution of particulate matter concentrations (PM2.5 and PM10) reported in Figure 7 and Figure 8, it is possible to identify several days where the PM exceeded the daily limit and in general two periods of persistence of PM in the air, between 16 February and 25 February and between 17 March and 20 March, respectively. These periods of persistence mainly affected the provinces of Bergamo, Brescia, Cremona, Lodi, Mantova, Milano, and Pavia. Conversely, Como, Lecco, Monza, Sondrio, and Varese did not present periods of long persistence of particulate matter. It is worth noting that the provinces with the highest rate of persistence of particulate matter coincide with the provinces that present the highest number of infected people.

A lag analysis of the correlation between PMs concentration and the incidence rate is carried out by calculating the cross-correlations obtained shifting the two series through time. After deriving the cross-correlation values between the two series, the time-delay (τ) where the two series best fit can be obtained by the argument of the maximum of the cross-correlation:

$$\tau =\mathrm{argmax}\left(\left(f\times g\right)\left(t\in \mathbb{R}\right)\right)$$

where f ad g denote the two time series considered, while t∈ℝ represents the time span. The results reported in Table 2 and Table 3 present the highest correlation values with delays between 10 and 11 days. In a similar way, Table 4 reports the cross-correlation between the incidence rate and the NAA parameter. The values obtained show that the net atmospheric acidity, contributing to the preservation of the vitality of the virus, presents a good correlation with the incidence rate with a time delay between eight and 11 days.

Another analysis can be carried out considering the wind speed that may have contributed to the spreading of aerosolized virus through the air. Among the days that presented strong wind speed (>5 m/s), the period between 10 February and 11 February just followed a period where most of the survival conditions were present. It is also interesting to note that on 26 February, a strong wind day followed a period of particulate matter persistence. In both cases, this occurrence may allow the removal of fine particulate and at the same time the resuspension of coarse PM.

**Figure 9:**
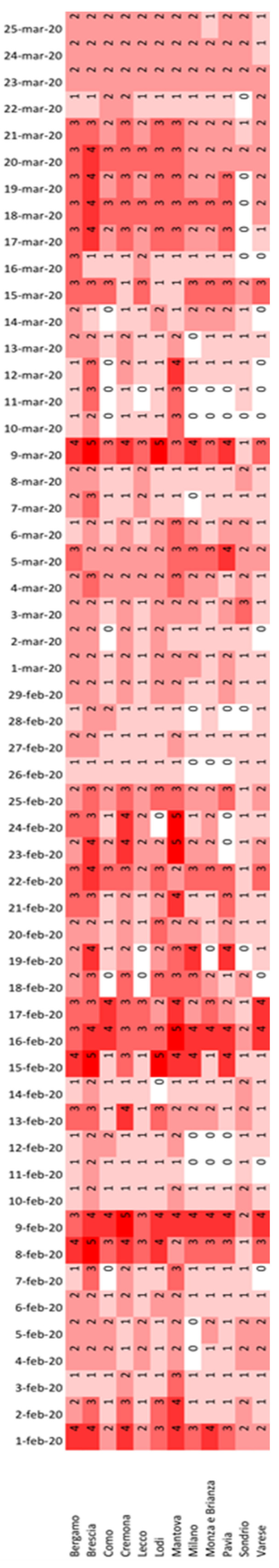
Co-occurrence of PM10, PM2.5, relative humidity, ambience temperature, and net atmospheric acidity.

In Figure 9, the co-occurrence of PM10, PM2.5, relative humidity, ambient temperature, and net atmospheric acidity (NAA) has been reported. In particular, for each province, for each day, the figure reports values between 0 and 5, corresponding to the verification of one or more of the following conditions:

- PM10 > 50 μg/m^3^
- PM2.5 > 50 μg/m^3^
- 75% < RH < 85%
- 4 °C < AT < 8 °C
−0.5 < NAA < 0.5

The results show five main periods where most of the above-mentioned conditions were satisfied. These periods are 8–9 February, 15–16 February, 22–25 February, 9 March, and 18 March. Comparing the three central periods to the insurgence of the symptoms reported in Figure 6, it is possible to note that there is a regular time-shift of approximately ten days with the three peaks (29 February, 9 March, and 19 March) in the insurgence of the symptoms reported in Figure 6. It is important to note that this period is compatible with the average incubation period of the SARS-CoV-2 virus.

## 4. Discussion

Our results showed a positive correlation between the peak infectiousness of SARS-CoV-2 for 29 February, 9 March, and 19 March and a given combination of climate elements and air pollution levels. This correlation was confirmed for the same days in all the 12 provinces of the Lombardy region. To explain these results, it can be assumed that viral respiratory diseases have a multifactorial nature; namely, they are the consequence of a complex combination of different chemical, physical, and biological factors that result in the features of the outbreak [52], referred to hereafter as “net effect”. These factors include individual host factors like the status of the host immune system that can regulate the progression from infection to disease. Innate immune system is the very first line of defense against infections, but only limited information is available so far about the host innate immune status of SARS-CoV-2 infected patients [53]. Individual behavioral factors like tobacco smoking and diet can have important immunomodulatory effects in humans, thus influencing the host susceptibility to viral infections and outbreak of chronic respiratory diseases [54,55].

A second group of factors is atmospheric environment factors that (i) can increase the organism’s vulnerability, thus representing risk factors for chronic respiratory diseases, and (ii) and affect the steps of airborne viral transmission. These steps are:

(i). Droplets emission and genesis of viral bioaerosols,
(ii). Transmission and diffusion of viral bioaerosols,
(iii). Retention of infectivity over time, and
(iv). Probability of infection for a given exposure dose.

All viruses are organized macromolecular entities without metabolism and repair mechanisms: SARS-CoV-2 possess a nucleocapsid and RNA genome surrounded by an external lipid envelope. This concept is fundamental for understanding the higher impact that chemical and physical atmospheric environment factors could exert on airborne viruses.

However, the atmospheric processes constitute a dynamic complex system characterized by several interdependent and independent factors, including gaseous composition, pollution levels, and climate elements like temperature and relative humidity. The understanding of nonlinear and hardly standardizable interactions between these factors is a key concept to explain differences in COVID-19 outbreaks occurring in different countries characterized by very different pollution levels and population densities compared to Wuhan area. Thus, the different chemical and physical factors of atmospheric processes can have a different weight concerning the “net effect”, depending on how they change at the local or regional level. Against this breakdown, it would also be important to assess the extreme conditions, e.g., of air pollution levels vs. contagiousness of a virus and the disease outbreak. In addition, net effect can show different and hardly predictable additive, antagonistic, or synergistic features.

Air pollutants like particulate matter (PM10 and PM2.5) are widely recognized as significant risk factors for respiratory disease-related morbidity and mortality worldwide [56]. Some studies also considered the effects of PM and other air pollutants in combination with specific values of relative humidity and temperature [57,58] or risk factors associated to the chemical composition of PM [59]. The impairment of immune functions as a consequence of exposure to PM is well documented [56] and the chronic exposure to atmospheric contamination (including PM) may lead to pro-inflammatory responses and high incidence of respiratory and cardiac affections, thus favoring the spread of the SARS-CoV-2 [60,61,62,63,64].

A presumptive role of PM as carrier of SARS-CoV-2 have been recently suggested. In some works, the concentration and the presence of airborne SARS-COV-2 RNA have been measured in sampled outdoor and indoor aerosols and in PM10 of outdoor air samples [65,66]. To date, no information is available about real stability and virulence of SARS-CoV-2 viral particles associated with PM or aerosol. Further studies should be carried out to assess stability and virulence of SARS-CoV-2 viral particles associated to PM and/or aerosol. The condition of indoor environments and the relationships with outdoor air pollution should be also considered if PM is supposed to have a role as carrier of viral particles. More stable environmental conditions, air stagnation, as well as limited dispersion and higher persistence of air pollutants like PM may indeed improve the stability of viral particles in indoor environments [67,68]. For the period considered in our work, indoor pollution in combination with behavioral habits during wintertime, when people spend most of their time indoors, may have had a role in the SARS-CoV2 infection spread.

Together with PM10 and PM2.5, possible relationships between outdoor air pollution and COVID-19 outbreak have been recently reported in literature for different parameters: O_3_, SO_2_, NO, NO_2_, and CO [21,69,70,71].

However, none of these works have considered the possible role of ammonia (NH_3_). Ammonia is an alkaline gas that drives the formation of major components of inorganic secondary PM (NH_4_)2SO_4_, NH_4_HSO_4_ and NH_4_NO_3_ [72] and exerts a considerable effect on pH of atmospheric water [73].

The NH_3_ values reported would lead to atmospheric pH around neutrality or slightly acidic or basic conditions. This occurrence was frequently recorded in each province within Lombardy region during the period considered (8–9 February, 15–16 February, and 9 March). It can be hypothesized that these NAA values can affect net surface charge of airborne viral particles with presumptive “protection’” from pH-dependent tertiary/secondary structure denaturation through hydrolytic and oxidative reactions. Net surface charge strongly influences colloidal behavior of viral particles, virus sorption processes, and likelihood of virus attachment through adhesion forces between virus particles and charged surface of host cells [74,75]. Therefore, the NAA may has been an important factor in the “net effect” in the case study described in this work.

## 5. Conclusions

Our results showed that the air pollution and the climate conditions might promote the spread of active viral particles. The understanding of complex interaction between different chemical, physical, and biological factors, which can lead to the development of disease outbreaks (“net effect”), is of utmost importance for addressing the future research, but also for planning the development and management of interventions to contain future spread of viral infections. These aspects could have also important implications in the public health management both to convey and improve receptivity of health-related communications and dissemination to general population, and to define more effective prevention strategies.

## Figures and Tables

**Table 1:** Pearson correlation coefficients evaluating the correlation between the prevalence infected population and air pollutants average concentrations, respectively.

*ρ*	PM10	PM2.5	O_3_	NO_2_	NO	NH_3_	CO	SO_2_	NAA
**Prevalence of infected population**	0.54	0.51	−0.03	0.31	0.26	0.75	0.51	0.05	0.27
**Z-Score**	0.75	0.76	0.78	0.79	0.72	0.82	0.81	0.82	0.87

**Table 2:** Cross-correlation values between the daily incidence of infection and the PM2.5 concentration obtained varying the lag between the two series.

Lag (Days)	8	9	10	11	12	13	14
Bergamo	−0.13	0.20	0.45	0.26	0.20	−0.14	−0.40
Brescia	−0.02	0.07	0.28	0.34	0.26	−0.15	−0.43
Como	−0.24	0.25	0.60	0.11	0.01	−0.13	−0.35
Cremona	−0.09	0.08	0.28	0.29	0.28	−0.12	−0.40
Lecco	−0.25	0.21	0.56	0.25	0.03	−0.14	−0.38
Lodi	−0.09	0.12	0.28	0.17	0.20	−0.13	−0.40
Mantova	0.00	0.03	0.31	0.39	0.30	−0.15	−0.38
Milano	−0.17	0.20	0.49	0.15	−0.04	−0.22	−0.41
Monza	−0.20	0.23	0.59	0.24	−0.04	−0.19	−0.37
Pavia	−0.07	0.27	0.27	0.03	−0.03	−0.21	−0.31
Sondrio	−0.31	−0.05	0.27	0.09	0.28	0.02	−0.21
Varese	−0.30	0.20	0.57	0.13	0.08	−0.15	−0.37

**Table 3:** Cross-correlation values between the daily incidence of infection and the PM10 concentration obtained varying the lag between the two series.

Lag (Days)	8	9	10	11	12	13	14
Bergamo	−0.15	0.20	0.47	0.28	0.20	−0.14	−0.40
Brescia	−0.14	0.09	0.43	0.35	0.30	−0.13	−0.42
Como	−0.30	0.20	0.53	0.09	0.11	−0.12	−0.34
Cremona	−0.05	0.12	0.30	0.26	0.27	−0.14	−0.43
Lecco	−0.30	0.23	0.52	0.16	0.14	−0.13	−0.36
Lodi	−0.09	0.17	0.27	0.17	0.19	−0.16	−0.42
Mantova	−0.04	0.02	0.33	0.41	0.34	−0.18	−0.38
Milano	−0.18	0.22	0.54	0.17	−0.01	−0.23	−0.43
Monza	−0.21	0.24	0.61	0.25	−0.04	−0.22	−0.38
Pavia	−0.09	0.29	0.33	0.06	0.04	−0.19	−0.36
Sondrio	−0.33	−0.09	0.32	0.11	0.24	−0.01	−0.21
Varese	−0.32	0.17	0.61	0.15	0.06	−0.18	−0.38

**Table 4:** Cross-correlation values between the daily incidence of infection and the NAA values obtained varying the lag between the two series.

Lag (Days)	8	9	10	11	12	13	14
Bergamo	0.35	0.09	0.09	0.10	0.14	0.16	0.03
Brescia	0.40	0.04	−0.12	−0.19	−0.01	0.09	0.20
Como	−0.19	0.21	0.56	0.30	0.07	−0.09	−0.26
Cremona	0.36	−0.04	−0.18	−0.18	0.00	0.18	0.15
Lecco	−0.11	0.16	0.33	0.45	0.36	−0.02	−0.28
Lodi	0.24	0.01	−0.03	−0.04	0.00	0.13	−0.04
Mantova	0.21	−0.09	−0.19	−0.14	0.14	0.07	0.23
Milano	0.04	0.10	0.32	0.16	−0.03	−0.06	−0.11
Monza	0.05	0.25	0.38	0.33	0.13	−0.06	−0.13
Pavia	0.07	0.10	0.05	−0.04	0.04	−0.18	−0.17
Sondrio	−0.11	0.26	0.36	0.16	0.39	0.00	−0.17
Varese	−0.15	0.21	0.45	0.03	−0.04	−0.08	−0.19

## Data Availability

Publicly available datasets were analyzed in this study. The data were collected through the Copernicus Atmosphere Monitoring Service, implemented by the European Centre for Medium-Range Weather Forecasts as part of the Copernicus Programme. These data can be found here: http://atmosphere.copernicus.eu/; http://ecmwf.int/; http://copernicus.eu/.
